# Exact Solutions for the Integrable Sixth-Order Drinfeld-Sokolov-Satsuma-Hirota System by the Analytical Methods

**DOI:** 10.1155/2014/840689

**Published:** 2014-09-09

**Authors:** Jalil Manafian Heris, Mehrdad Lakestani

**Affiliations:** Department of Applied Mathematics, Faculty of Mathematics Science, University of Tabriz, 29 Bahman Boulevard, Tabriz 5166616471, Iran

## Abstract

We establish exact solutions including periodic wave and solitary wave solutions for the integrable sixth-order Drinfeld-Sokolov-Satsuma-Hirota system. We employ this system by using a generalized (*G*′/*G*)-expansion and the generalized tanh-coth methods. These methods are developed for searching exact travelling wave solutions of nonlinear partial differential equations. It is shown that these methods, with the help of symbolic computation, provide a straightforward and powerful mathematical tool for solving nonlinear partial differential equations.

## 1. Introduction

The general form of the Drinfeld-Sokolov-Satsuma-Hirota system that is going to be studied in this paper is given by

(1) $$\begin{array}{c} w_{t}-6ww_{x}+w_{xxx}-6v_{x}=0,\\ v_{t}-2v_{xxx}+6wv_{x}=0, \end{array}$$

which was developed in [1] as one example of nonlinear equations possessing Lax pairs of a special form [2–4]. System (1) was independently presented by Drinfeld and Sokolov [1] and Satsuma and Hirota [5]. However, this system was found as a special case of the four-reduction of the KP hierarchy [2, 5]. Wazwaz [6] studied this system by using Hirota's bilinear, the tanh-coth, the tan-cot, and the Exp-function methods. In [2], the truncated singular expansions method was used to construct an explicit Bäcklund transformation method to derive special solutions of this equation. Also, in [3], the sine-cosine method and the tanh method were used to obtain exact travelling wave solutions. Recently, the investigation of exact travelling wave solutions to nonlinear partial differential equations plays an important role in the study of nonlinear modelling physical phenomena. The study of the travelling wave solutions plays an important role in nonlinear sciences. Up to now, there exist many powerful methods to construct exact solutions of nonlinear differential equations. A variety of powerful methods have been presented, such as Hirota's bilinear method [7–10], the inverse scattering transform [11], sine-cosine method [12], homotopy perturbation method [13], homotopy analysis method [14, 15], variational iteration method [16, 17], Bäcklund transformation [18, 19], Exp-function method [17, 20, 21], (*G*′/*G*)-expansion method [22, 23], Laplace Adomian decomposition method [24], and differential transform method [25]. Here, we use an effective method for constructing a range of exact solutions for following nonlinear partial differential equations that were first proposed by Wang et al. [26] which a new method called the (*G*′/*G*)-expansion method to look for travelling wave solutions of NLEEs. Zhang et al. [27] examined the generalized (*G*′/*G*)-expansion method and its applications. Authors of [28] used mKdV equation with variable coefficients using the (*G*′/*G*)-expansion method. Also, Bekir [23] used application of the (*G*′/*G*)-expansion method for nonlinear evolution equations. In this paper we explain the method which is called the (*G*′/*G*)-expansion method to look for travelling wave solutions of nonlinear evolution equations.

The paper is organized as follows. In Section 2, we briefly give the steps of the methods and apply the methods to solve the nonlinear partial differential equations. In Section 3 the application of generalized tanh-coth method to the DSSH equation will be introduced briefly, respectively. Also a conclusion is given in Section 4. Finally some references are given at the end of this paper.

## 2. Basic Ideas of the (*G*′/*G*)-Expansion and the Tanh-Coth Methods

### 2.1. The (*G*′/*G*)-Expansion Method

Another powerful analytical method is called (*G*′/*G*)-expansion method; we give the detailed description of the method which was first presented by Wang et al. [26].

**Step 1 .**

For a given NLPDE with independent variables *X* = (*x*, *y*, *z*, *t*) and dependent variable *u*,

(2) P(utt,utx,uty,utz,…u,ut,ux,uy,uz,uxx,uyy,uzz,uxy,000utt,utx,uty,utz,…)=0

can be converted to ODE:

(3) $$\begin{array}{c} M\left(u,-cu^{\prime },u^{\prime },u^{\prime },u^{\prime \prime },\ldots \right)=0, \end{array}$$

where transformation *ξ* = *x* + *y* − *ct* is wave variable. Also, *c* is a constant to be determined later.

**Step 2 .**

We seek its solutions in the more general polynomial form as follows:

(4) $$\begin{array}{c} u\left(\xi \right)=a_{0}+\overset{m}{\underset{k=1}{\sum }}a_{k}{\left(\frac{G^{\prime }(\xi )}{G(\xi )}\right)}^{k}, \end{array}$$

where *G*(*ξ*) satisfies the second order LODE in the following form:

(5) $$\begin{array}{c} G^{\prime \prime }\left(\xi \right)+\lambda G^{\prime }\left(\xi \right)+\mu G\left(\xi \right)=0, \end{array}$$

where *a*_0_, *a*_*k*_ (*k* = 1,2,…, *m*), *λ*, and *μ* are constants which will be determined later, *a*_*m*_ = 0, but the degree of which is generally equal to or less than *m* − 1; the positive integer *m* can be determined by considering the homogeneous balance between the highest order derivatives and nonlinear terms appearing in (3).

**Step 3 .**

Substituting (4) and (5) into (3) with the value of *n* obtained in Step 1, collecting the coefficients of (*G*′(*ξ*)/*G*(*ξ*))^*k*^ (*k* = 0,1, 2,…), and then setting each coefficient to zero, we can get a set of overdetermined partial differential equations for *a*_0_, *a*_*i*_ (*i* = 1,2,…, *n*), *λ*, *c*, and *μ* with the aid of symbolic computation Maple.

**Step 4 .**

Solving the algebraic equations in Step 3 and then substituting *a*_*i*_,…, *a*_*m*_, *c* and general solutions of (5) into (4) we can obtain a series of fundamental solutions of (2) depending of the solution *G*(*ξ*) of (5).

### 2.2. The Generalized Tanh-Coth Method

We now describe the generalized tanh-coth method for the given partial differential equations. We give the detailed description of the method which to use this method; we take the following steps.

**Step 1 .**

For a given NLPDE with independent variables *X* = (*x*, *y*, *z*, *t*) and dependent variable *u*, we consider a general form of nonlinear equation

(6) P(u,ut,ux,uy,uz,uxx,uyy,uzz,000uxy,utt,utx,uty,utz,…)=0,

which can be converted to ODE

(7) $$\begin{array}{c} Q\left(u,-cu^{\prime },u^{\prime },u^{\prime },u^{\prime },u^{\prime \prime },\ldots \right)=0, \end{array}$$

where transformation *ξ* = *x* + *y* − *ct* is wave variable. Also, *c* is constant to be determined later.

**Step 2 .**

We introduce the Riccati equation as follows:

(8) $$\begin{array}{c} \Phi^{\prime }=r+p\Phi +q\Phi^{2},\;\;\Phi =\Phi \left(\xi \right),\;\;\xi =x-ct, \end{array}$$

which leads to the change of derivatives

(9) ddξ=(r+pΦ+qΦ2)ddΦ,d2dξ2=(r+pΦ+qΦ2)×[(p+2qΦ)ddΦ+(r+pΦ+qΦ2)d2dΦ2],d3dξ3=(r+pΦ+qΦ2)×[d2dΦ2+(r+pΦ+qΦ2)2d3dΦ3(6q2Φ2+6pqΦ+2rq+p2)ddΦ00 +(6q2Φ3+9pqΦ2+3(p2+2rq)Φ+3rp)00 ×d2dΦ2+(r+pΦ+qΦ2)2d3dΦ3],

which admits the use of a finite series of functions of the form

(10) $$\begin{array}{c} u\left(\xi \right)=S\left(\Phi \right)=\overset{m}{\underset{k=0}{\sum }}a_{k}\Phi^{k}+\overset{m}{\underset{k=1}{\sum }}b_{k}\Phi^{-k}, \end{array}$$

where *a*_*k*_ (*k* = 0,2,…, *m*), *b*_*k*_ (*k* = 1,2,…, *m*), *p*, *r*, and *q* are constants to be determined later. But, the positive integer *m* can be determined by considering the homogeneous balance between the highest order derivatives and nonlinear terms appearing in (7). If *m* is not an integer, then a transformation formula should be used to overcome this difficulty. For aforementioned method, expansion (10) reduces to the standard tanh method [29] for *b*_*k*_ = 0, 1 ≤ *k* ≤ *m*.

**Step 3 .**

Substituting (8) and (9) into (7) with the value of *m* obtained in Step 2, collecting the coefficients of Φ^*k*^ (*k* = 0,1, 2,…), and then setting each coefficient to zero, we can get a set of overdetermined partial differential equations for *a*_0_, *a*_*i*_ (*i* = 1,2,…, *m*), *b*_*i*_ (*i* = 1,2,…, *m*) *p*, *q*, and *r* with the aid of symbolic computation Maple.

**Step 4 .**

Solve the algebraic equations in Step 3 and then substitute *a*_0_, *a*_1_, *b*_1_,…, *a*_*m*_, *b*_*m*_, *c* in (10).

**Step 5 .**

We will consider that the following twenty seven solutions of generalized Riccati differential equation (8) are given in [30–32].

**Case 1 .**

For each *pq* ≠ 0 or *qr* ≠ 0 and Δ = *p* ^2^ − 4*qr* > 0, (8) has the following solutions:

(11) Φ1(ξ)=−12q[p+Δtanh(Δξ2)],Φ2(ξ)=−12q[p+Δcoth⁡(Δξ2)],Φ3(ξ)=−12q[p+Δ[tanh(Δξ)±i sech(Δξ)]],00000000000000000000000000000i=−1,Φ4(ξ)=−12q[p+Δ[coth⁡(Δξ)±csch(Δξ)]],Φ5(ξ)=−14q[2p+Δ[tanh(Δξ4)±coth⁡(Δξ4)]],Φ6(ξ)=12q[−p+(A2+B2)Δ−AΔcosh⁡(Δξ)Asinh⁡(Δξ)+B],Φ7(ξ)=12q[−p−(B2−A2)Δ+AΔcosh⁡(Δξ)Asinh⁡(Δξ)+B],

where *A* and *B* are two nonzero real constants and satisfy *B* ^2^ − *A* ^2^ > 0:

(12) Φ8(ξ)=2rcosh⁡(Δξ/2)Δsinh⁡(Δξ/2)−pcosh⁡(Δξ/2),Φ9(ξ)=−2rsinh⁡(Δξ/2)psinh⁡(Δξ/2)−Δcosh⁡(Δξ/2),Φ10(ξ)=2rcosh⁡(Δξ/2)Δsinh⁡(Δξ)−pcosh⁡(Δξ)±iΔ,Φ11(ξ)=2rsinh⁡(Δξ/2)−psinh⁡(Δξ)+Δcosh⁡(Δξ)±Δ,Φ12(ξ)=(4rsinh⁡(Δξ/4)cosh⁡(Δξ/4))×(−2psinh⁡(Δξ/4)cosh⁡(Δξ/4)000+2Δ cosh⁡2(Δξ/4)−Δ)−1.

**Case 2 .**

For each *pq* ≠ 0 or *qr* ≠ 0 and Δ = *p* ^2^ − 4*qr* < 0, (8) has the following solutions:

(13)  Φ13(ξ)=−12q[p−−Δtan(−Δξ2)], Φ14(ξ)=−12q[p+−Δcot(−Δξ2)], Φ15(ξ)=−12q[p−−Δ[tan(−Δξ)±sec(−Δξ)]], Φ16(ξ)=−12q[p+−Δ[cot(−Δξ)±csc(−Δξ)]],Φ17(ξ) =−14q[2p−−Δ[tan(−Δξ4)−cot(−Δξ4)]],Φ18(ξ) =12q[−p+±(A2+B2)Δ−A−Δcos⁡(−Δξ)Asin(−Δξ)+B],Φ19(ξ) =12q[−p−±(B2−A2)Δ+A−Δcos⁡(−Δξ)Asin(−Δξ)+B],

where *A* and *B* are two nonzero real constants and satisfy *B* ^2^ − *A* ^2^ > 0:

(14)  Φ20(ξ)=−2rcos⁡(−Δξ/2)−Δsin(−Δξ/2)+pcos⁡(−Δξ/2), Φ21(ξ)=2rsin(−Δξ/2)−psin(−Δξ/2)+−Δcos⁡(−Δξ/2), Φ22(ξ)=−2rcos⁡(−Δξ/2)−Δsin(−Δξ)+pcos⁡(−Δξ)±−Δ, Φ23(ξ)=2rsin(−Δξ/2)−psin(−Δξ)+−Δcos⁡(−Δξ)±−Δ,Φ24(ξ)=(4rsin(−Δξ/4)cosh⁡(−Δξ/4)).00000000×(−2psin(−Δξ/4)cos⁡(−Δξ/4)00000000000+2−Δcos⁡2(−Δξ/4)−−Δ)−1.

**Case 3 .**

For *r* = 0 and *pq* ≠ 0 (8) has the following solutions:

(15) Φ25(ξ)=−pdq[d+cosh⁡(pξ)−sinh⁡(pξ)],Φ26(ξ)=p[cosh⁡(pξ)+sinh⁡(pξ)]q[d+cosh⁡(pξ)+sinh⁡(pξ)],

where *d* is an arbitrary constant.

**Case 4 .**

For *r* = *p* = 0 and *q* ≠ 0 (8) has the following solution:

(16) $$\begin{array}{c} \Phi_{27}\left(\xi \right)=\frac{-1}{q\xi +c}, \end{array}$$

where *c* is an arbitrary constant.

But from (*G*′/*G*)-expansion method, we have

(17) (G′G)′=G′′G−G′2G2=(−λG′−μG)G−G′2G2=−μ−λ(G′G)−(G′G)2;

we set *F* = *G*′/*G* and then

(18) $$\begin{array}{c} F^{\prime }=-\mu -\lambda F-F^{2}. \end{array}$$

Thus we obtain that the exact solutions derived by (*G*′/*G*)-expansion are same as ones by the generalized tanh-coth methods. Hence we use only the generalized tanh-coth method.

## 3. Application of the Generalized Tanh-Coth Method

We next consider DSSH equation with the generalized tanh-coth method in the following form:

(19) $$\begin{array}{c} w_{t}-6ww_{x}+w_{xxx}-6v_{x}=0,\\ v_{t}-2v_{xxx}+6wv_{x}=0. \end{array}$$

Proceeding as before we get

(20) $$\begin{array}{c} 2u^{\left(v\right)}-18u^{\prime }u^{\prime \prime \prime }-9{\left(u^{\prime \prime }\right)}^{2}+12{\left(u^{\prime }\right)}^{3}-c^{2}u^{\prime }-cu^{\prime \prime \prime }=0. \end{array}$$

In order to determine value of *m*, we balance *u* ^(*v*)^ with *u*′*u*′′′ in (20), and by using (10) we obtain *m* = 1. We can suppose that the solutions of (19) are of the following form:

(21) $$\begin{array}{c} u\left(\xi \right)=a_{0}+a_{1}\Phi +\frac{b_{1}}{\Phi }. \end{array}$$

Substituting (21) into (20) and by using the well-known software Maple, we obtain the system of the following results:

(22) $$\begin{array}{c} a_{0}=2q,\;\;a_{1}=2q,\;\;b_{1}=0,\;\;c=p^{2}-4qr, \end{array}$$

or

(23) $$\begin{array}{c} a_{0}=2r,\;\;a_{1}=0,\;\;b_{1}=-2r,\;\;c=p^{2}-4qr, \end{array}$$

where *p*, *q*, *r*, and *c* are arbitrary constants. Substituting (22) and (23) into expression (21) we obtain

(24) $$\begin{array}{c} u\left(\xi \right)=2q+2q\Phi \left(\xi \right),\;\;\xi =x-\left(p^{2}-4qr\right)t, \end{array}$$

(25) $$\begin{array}{c} u\left(\xi \right)=2r-\frac{2r}{\Phi \left(\xi \right)},\;\;\xi =x-\left(p^{2}-4qr\right)t. \end{array}$$

By the manipulation as explained in the previous section, we have the following.

*(I) The First Set for *(24). By using Case 1 from Section 2 we have

(26) u1(x,t)=2q{1−12q[p+Δtanh(Δξ2)]},u2(x,t)=2q{1−12q[p+Δcoth⁡(Δξ2)]},u3(x,t) =2q{1−12q[p+Δ[tanh(Δξ)±i sech(Δξ)]]},u4(x,t) =2q{1−12q[p+Δ[coth⁡(Δξ)±csch(Δξ)]]},u5(x,t) =2q{1−14q0000l00l×[2p+Δ[tanh(Δξ4)00000000000000000l±coth⁡(Δξ4)]]},u6(x,t)=2q{1+12q[−p+(A2+B2)Δ−AΔcosh⁡(Δξ)Asinh⁡(Δξ)+B]},u7(x,t)=2q{1+12q[−p−(B2−A2)Δ+AΔcosh⁡(Δξ)Asinh⁡(Δξ)+B]},u8(x,t) =2q{1+2rcosh⁡(Δξ/2)Δsinh⁡(Δξ/2)−pcosh⁡(Δξ/2)},u9(x,t) =2q{1+−2rsinh⁡(Δξ/2)psinh⁡(Δξ/2)−Δcosh⁡(Δξ/2)},u10(x,t) =2q{1+2rcosh⁡(Δξ/2)Δsinh⁡(Δξ)−pcosh⁡(Δξ)±iΔ},u11(x,t) =2q{1+2rsinh⁡(Δξ/2)−psinh⁡(Δξ)+Δcosh⁡(Δξ)±Δ},u12(x,t)=2q{+2Δcosh⁡2(Δξ/4)−Δ)−1)1+(4rsinh⁡(Δξ/4)cosh⁡(Δξ/4))000000000000l×(−2psinh⁡(Δξ/4)cosh⁡(Δξ/4)00000000000000000+2Δcosh⁡2(Δξ/4)−Δ)−1}.

By using Case 2 from Section 2 we have

(27) u13(x,t)=2q{1−12q[p−−Δtan(−Δξ2)]},u14(x,t)=2q{1−12q[p+−Δ cot(−Δξ2)]},u15(x,t) =2q{1−12q[p−−Δ[tan(−Δξ)±sec(−Δξ)]]},u16(x,t) =2q{1−12q[p+−Δ[cot(−Δξ)±csc(−Δξ)]]},u17(x,t) =2q{1−14q000l000×[2p−−Δ[tan(−Δξ4)000000000000000000l−cot(−Δξ4)]]},u18(x,t) =2q{1+12q000l000×[1+12q−p+(±(A2+B2)Δ−A−Δcos⁡(−Δξ))0000000000 ×(Asin(−Δξ)+B)−11+12q]},u19(x,t)=2q{1+12q000l00×[1+12q−p−(±(B2−A2)Δ+A−Δcos⁡(−Δξ))000000000×(Asin(−Δξ)+B)−11+12q]},u20(x,t) =2q{1+−2rcos⁡(−Δξ/2)−Δsin(−Δξ/2)+pcos⁡(−Δξ/2)},u21(x,t) =2q{1+2rsin(−Δξ/2)−psin(−Δξ/2)+−Δcos⁡(−Δξ/2)},u22(x,t) =2q{1+−2rcos⁡(−Δξ/2)−Δsin(−Δξ)+pcos⁡(−Δξ)±−Δ},u23(x,t)=2q{1+2rsin(−Δξ/2)−psin(−Δξ)+−Δcos⁡(−Δξ)±−Δ},u24(x,t) =2q{+2−Δ cos⁡2(−Δξ4)−−Δ)−11+(4rsin(−Δξ4)cosh⁡(−Δξ4))0000000l0000×(−2psin(−Δξ4)cos⁡(−Δξ4)000000000000+2−Δ cos⁡2(−Δξ4)−−Δ)−1}.

By using Case 3 from Section 2 we have

(28) $$\begin{array}{c} u_{25}\left(x,t\right)=2q\left\{1+\frac{-pd}{q\left[d+\cosh\left(p\xi \right)-\sinh\left(p\xi \right)\right]}\right\},\\ u_{26}\left(x,t\right)=2q\left\{1+\frac{p\left[\cosh\left(p\xi \right)+\sinh\left(p\xi \right)\right]}{q\left[d+\cosh\left(p\xi \right)+\sinh\left(p\xi \right)\right]}\right\}. \end{array}$$

By using Case 4 from Section 2 we have

(29) $$\begin{array}{c} u_{27}\left(x,t\right)=2q\left\{1-\frac{1}{q\xi +c}\right\}, \end{array}$$

where *ξ* = *x* − (*p* ^2^ − 4*qr*)*t*.

*(II) The Second Set for *(25). By using Case 1 from Section 2 we have

(30)  u1(x,t)=2r{1+2q[p+Δtanh(Δξ/2)]}, u2(x,t)=2r{1+2q[p+Δcoth⁡(Δξ/2)]},u3(x,t) =2r{1+2q[p+Δ[tanh(Δξ)±i sech(Δξ)]]},u4(x,t) =2r{1+2q[p+Δ[coth⁡(Δξ)±csch(Δξ)]]},u5(x,t) =2r{±coth⁡(Δξ4)]]−11+4q000000l×[2p+Δ[tanh(Δξ4)000000000000000000±coth⁡(Δξ4)]]−1},u6(x,t)=2r{[−p+(((A2+B2)Δ−AΔcosh⁡(Δξ))1−2q000l00 ×[−p+(((A2+B2)Δ−AΔcosh⁡(Δξ))0000000000000000+(Asinh⁡(Δξ)+B)−1)−p+(((A2+B2)Δ−AΔcosh⁡(Δξ))]−1},u7(x,t)=2r{[(Asinh⁡(Δξ)+B)−1]−11−2q000l00×[−p−((B2−A2)Δ+AΔcosh⁡(Δξ))000000000×(Asinh⁡(Δξ)+B)−1]−1},u8(x,t) =2r{1−Δsinh⁡(Δξ/2)−pcosh⁡(Δξ/2)2rcosh⁡(Δξ/2)},u9(x,t) =2r{1+psinh⁡(Δξ/2)−Δcosh⁡(Δξ/2)2rsinh⁡(Δξ/2)},u10(x,t) =2r{1−Δsinh⁡(Δξ)−pcosh⁡(Δξ)±iΔ2rcosh⁡(Δξ/2)},u11(x,t) =2r{1−−psinh⁡(Δξ)+Δcosh⁡(Δξ)±Δ2rsinh⁡(Δξ/2)},u12(x,t)=2r{(4rsinh⁡(Δξ4)cosh⁡(Δξ4))−11−(−2psinh⁡(Δξ4)cosh⁡(Δξ4)000000l0000+2Δ cosh⁡2(Δξ4)−Δ)0000000×(4rsinh⁡(Δξ4)cosh⁡(Δξ4))−1}.

By using Case 2 from Section 2 we have

(31) u13(x,t)=2r{1+2q[p−−Δtan(−Δξ/2)]},u14(x,t)=2r{1+2q[p+−Δ cot(−Δξ/2)]},u15(x,t) =2r{1+2q[p−−Δ[tan(−Δξ)±sec(−Δξ)]]},u16(x,t) =2r{1+2q[p+−Δ[cot(−Δξ)±csc(−Δξ)]]},u17(x,t)=2r{1+4q[2p−−Δ[tan(−Δξ/4)−cot(−Δξ/4)]]},u18(x,t)=2r{(Asin(−Δξ)+B)−1]−11−2q000l00×[−p+(±(A2+B2)Δ−A−Δcos⁡(−Δξ))000000000×(Asin(−Δξ)+B)−1]−1},u19(x,t)=2r{×(Asin(−Δξ)+B)−1]−11−2q00l00×[−p−(±(B2−A2)Δ+A−Δcos⁡(−Δξ))00000000×(Asin(−Δξ)+B)−1]−1},u20(x,t) =2r{1+−Δsin(−Δξ/2)+pcos⁡(−Δξ/2)2rcos⁡(−Δξ/2)},u21(x,t) =2r{1−−psin(−Δξ/2)+−Δcos⁡(−Δξ/2)2rsin(−Δξ/2)},u22(x,t) =2r{1+−Δsin(−Δξ)+pcos⁡(−Δξ)±−Δ2rcos⁡(−Δξ/2)},u23(x,t) =2r{1−−psin(−Δξ)+−Δcos⁡(−Δξ)±−Δ2rsin(−Δξ/2)},u24(x,t) =2r{(4rsin(−Δξ4)cosh⁡(−Δξ4))−11−(−2psin(−Δξ4)cos⁡(−Δξ4)000000l0000+2−Δcos⁡2(−Δξ4)−−Δ)0000000×(4rsin(−Δξ4)cosh⁡(−Δξ4))−1}.

By using Case 3 from Section 2 we have

(32) $$\begin{array}{c} u_{25}\left(x,t\right)=2r\left\{1+\frac{q\left[d+\cosh\left(p\xi \right)-\sinh\left(p\xi \right)\right]}{pd}\right\},\\ u_{26}\left(x,t\right)=2r\left\{1-\frac{q\left[d+\cosh\left(p\xi \right)+\sinh\left(p\xi \right)\right]}{p\left[\cosh\left(p\xi \right)+\sinh\left(p\xi \right)\right]}\right\}. \end{array}$$

By using Case 4 from Section 2 we have

(33) $$\begin{array}{c} u_{27}\left(x,t\right)=2r\left\{1+q\xi +c\right\}, \end{array}$$

where *ξ* = *x* − (*p* ^2^ − 4*qr*)*t*. By using the *w*(*x*, *t*) = *u*_*x*_(*x*, *t*), *v*(*x*, *t*) = (1/6)(*u*_*t*_ − 3(*u*_*x*_)^2^ + *u*_*xxx*_), can be used to get the solutions of the DSSH system (19). It can be seen that the results are the same, with comparing results in the literature [6]. We obtained analytical solutions by the generalized (*G*′/*G*)-expansion and the generalized tanh-coth methods. Also, in this paper we can see correlation between (*G*′/*G*)-expansion method and tanh-coth methods. We have succeeded in identifying the equivalence of the two methods under special conditions [33]. Consequently, the solution of the equations via (*G*′/*G*)-expansion method is exactly the same as the solution of tanh-coth method if the conditions are satisfied. In fact, we can see that the tanh-coth method is a special case of the (*G*′/*G*)-expansion method.

## 4. Conclusion

In this paper we investigated the Drinfeld-Sokolov-Satsuma-Hirota system by using the generalized (*G*′/*G*)-expansion and the generalized tanh-coth methods which are useful methods for finding travelling wave solutions of nonlinear evolution equations. These methods have been successfully applied to obtain some new generalized solitonary solutions to the DSSH equation. These exact solutions include three types: hyperbolic function solution, trigonometric function solution, and rational solution. The generalized (*G*′/*G*)-expansion method is more powerful in searching for exact solutions of NLPDEs. By comparing our results and Wazwaz's [6] results it can be seen that the results are the same. Also, new results are formally developed in this paper. It can be concluded that this method is a very powerful and efficient technique in finding exact solutions for wide classes of problems.
